# Pre-invasion history and demography shape the genetic variation in the insecticide resistance-related acetylcholinesterase 2 gene in the invasive Colorado potato beetle

**DOI:** 10.1186/1471-2148-13-13

**Published:** 2013-01-18

**Authors:** Saija Piiroinen, Leena Lindström, Anne Lyytinen, Johanna Mappes, Yolanda H Chen, Victor Izzo, Alessandro Grapputo

**Affiliations:** 1 Centre of Excellence in Biological Interactions Research, Department of Biological and Environmental Science, University of Jyväskylä, P.O. Box 35, FI-40014, Jyväskylä, Finland; 2 Department of Plant and Soil Sciences, University of Vermont, 63 Carrigan Drive, Burlington, VT, 05405, USA; 3 Department of Biology, University of Padua, Via U. Bassi, 58/B, 35121, Padua, Italy

**Keywords:** Acetylcholinesterase, Diapause protein 1, Invasion biology, Invasive species, Juvenile hormone esterase, Pesticide resistance, Resistance evolution, Selection

## Abstract

**Background:**

Invasive pest species offers a unique opportunity to study the effects of genetic architecture, demography and selection on patterns of genetic variability. Invasive Colorado potato beetle (*Leptinotarsa decemlineata*) populations have experienced a rapid range expansion and intense selection by insecticides. By comparing native and invasive beetle populations, we studied the origins of organophosphate (OP) resistance-associated mutations in the acetylcholinesterase 2 (*AChE2*) gene, and the role of selection and demography on its genetic variability.

**Results:**

Analysis of three Mexican, two US and five European populations yielded a total of 49 haplotypes. Contrary to the expectations all genetic variability was associated with a point mutation linked to insecticide resistance (S291G), this mutation was found in 100% of Mexican, 95% of US and 71% of European beetle sequences analysed. Only two susceptible haplotypes, genetically very differentiated, were found, one in US and one in Europe. The genetic variability at the *AChE2* gene was compared with two other genes not directly affected by insecticide selection, diapause protein 1 and juvenile hormone esterase. All three genes showed reduction in genetic variability indicative of a population bottleneck associated with the invasion.

**Conclusions:**

Stochastic effects during invasion explain most of the observed patterns of genetic variability at the three genes investigated. The high frequency of the S291G mutation in the *AChE2* gene among native populations suggests this mutation is the ancestral state and thus, either a pre-adaptation of the beetle for OP resistance or the *AChE2* is not the major gene conferring OP resistance. The long historical association with host plant alkaloids together with recombination may have contributed to the high genetic variation at this locus. The genetic diversity in the *AChE2* locus of the European beetles, in turn, strongly reflects founder effects followed by rapid invasion. Our results suggest that despite the long history of insecticide use in this species, demographic events together with pre-invasion history have been strongly influential in shaping the genetic diversity of the *AChE2* gene in the invasive beetle populations.

## Background

Genetic variability in a given gene is the result of a complex interplay of evolutionary forces such as selection, mutation rate and recombination, and demographic history of the populations including changes in population size, migration and divergence [1]. The evolution of insecticide resistance in invasive pest species offers an opportunity to study these forces where the form, target and history of selection, as well as invasion routes are often well known [2]. Evolution due to a strong directional selection by insecticides can be a rapid process [ [3] and references therein], usually involving only a few genes with pronounced phenotypic influences [4,5]. Typically, mutations that confer resistance are considered to occur at very low frequency in the population due to fitness costs in non-insecticide environments [2]. Strong insecticide selection subsequently leads to an increase in the resistance allele frequency and a general change in the allele composition of populations (e.g. [6]). Furthermore, a strong reduction in genetic variation is often observed as the originally rare mutation increases in frequency [7,8]. This reduction may occur not only in the locus targeted by selection but also in the surrounding areas through linkage and genetic hitchhiking [9,10]. Alternatively, selection can act on standing genetic variation (that is, the beneficial mutation has relatively high frequency) leading to soft sweeps or even maintain variability [11-13].

In addition to selection by insecticides, populations of invasive pest species are often affected by demographic events. For instance, genetic variation is often reduced by population bottlenecks and allele frequencies can be drastically changed by founder events [14]. Subsequent range expansions, typically observed in species invasions [15], can contribute to the spread of an insecticide resistance allele across large regions without local insecticide selection [16]. However, demographic events can be difficult to disentangle from selection as the former can lead to patterns of genetic variation similar to those caused by selection [1]. These confounding effects can, in principle, be separated by examining multiple loci since demographic history modifies the genetic pattern of the whole genome whereas only certain loci are affected by selection [1].

Studies of resistance genes have focused on revealing the nucleotide substitutions that are associated with resistance (e.g. [17,18]), while less attention has been paid to genetic variation in the resistance genes themselves. Yet the level and pattern of genetic variation at the gene and population level may give a more comprehensive picture of the evolutionary forces affecting populations [7,8,19]. Comparing populations before and after selection (or populations differing in their exposure to pesticide selection pressure) can provide important insights on the origins of resistance and how selection modifies patterns of genetic variability [10,13]. For instance, the Colorado potato beetle (*Leptinotarsa decemlineata*), an invasive insect pest of potato, is well known for its ability to evolve resistance to insecticides [20] and specific mutations at the target site of resistance to organophosphate and pyrethroids have been extensively investigated [21-26]. However, few studies have examined the population genetic history of agricultural insect pest populations (but see e.g. [13]). By examining the population genetic history of agricultural insect pests, it will provide insight as to whether insect pests appear to be preadapted to insecticide resistance, or whether resistance arises from *de novo* mutations under intense selection.

Organophosphates are one of the most common classes of insecticides used worldwide. They were introduced as insecticides in the 1930s after which their use increased rapidly and peaked in the 1970s and 1980s [27]. Due to intense use of OP insecticides, resistance to OPs subsequently appeared in various insect pest species, including the Colorado potato beetle as early as 1964 in US [3,28,29]. OP resistance in insects can be based on several mechanisms; the major ones are target-site insensitivity and enhanced detoxification of the toxicant. Also lowered availability of the toxicant may play a role in resistance (e.g. [5], see also [29]). Target-site insensitivity is caused by point mutations in two *AChE* genes (*AChE1* paralogous to and *AChE2* orthologous to *Drosophila melanogaster Ace* gene) [30] that encode for acetylcholinesterase enzymes that function in the synapses of the nervous system. These point mutations cause insensitivity at the target site of insecticides action (e.g. [20]). There are at least two point mutations in the *AChE2* gene that have been associated with OP resistance in the Colorado potato beetle [21,22]. The major resistance-conferring mutation, an amino acid substitution from serine to glycine (S291G), seems to be unique to this species [21]. The second mutation (arginine to lysine, R30K), may enhance the resistance conferred by the S291G [24].

In addition for being famous for its ability to evolve insecticide resistance, the invasion history of the Colorado potato beetle is relatively well known. The beetle is native to Mexico where it uses wild relatives of potato (*Solanum rostratum* Dunal*, S. angustifolium* Mill.) as host plants, and is not a pest of potato. It is thought that Spanish settlers that migrated northwards with cattle inadvertently carried *S. rostratum*, northwards. The beetle was first described feeding on *S. rostratum* in 1824 in Nebraska. The beetle became a serious pest after adapting to potato (*S. tuberosum*) in the American mid-west in the 1850s [31]. The first described outbreaks of the beetle occurred in 1859 in the Midwestern US [31]. Since, it has spread across the US and almost throughout Europe [32,33]. Although its establishment in Europe involved a founder event [34] which reduced neutral genetic variability, the beetle has retained a substantial amount of genetic variability in life history traits [33,35]. Currently, the Colorado potato beetle is considered to be the major threat to potato crops worldwide. If left uncontrolled, it can cause serious damage to potato crops [36]. The beetle’s complicated and versatile life history has contributed to its success. For instance, the beetle has a very high reproductive potential, both larvae and adults feed on potato, and adults can survive harsh winter conditions by overwintering in diapause burrowed into the soil [29,37].

We took advantage of the knowledge on one of the main mechanisms of OP resistance and combined it with the invasion history of the Colorado potato beetle to examine the origins of OP resistance-associated mutations, and the selective and demographic forces on the genetic variation in the *AChE2* gene using a population genetic approach. In order to disentangle the confounding effects of demography from selection, we examined genetic variation at two other nuclear genes, the diapause protein 1 (*DP1*) [38] and putative juvenile hormone esterase (*JHE*) [39,40]. If there was a clear sign of intense directional selection, we would expect lower genetic variability both at the *AChE2* locus than at the other two loci and in the invasive agricultural (US and Europe) than in native Mexican beetle populations. We would also expect a higher frequency of the OP resistance-associated S291G mutation in the invasive than in the native populations. Finally, we searched for evidence of population genetic structure at the loci among geographic regions and investigated if nucleotide variability at the *AChE2*, *DP1* and *JHE* genes are compatible with neutral expectations.

## Results

### Haplotypes and the frequency of resistance-associated mutations at the *AChE* gene

The *AChE2* sequences from the 98 Colorado potato beetles (10 populations, Europe: Finland, Russia, Estonia, Poland, Italy; US: Colorado, Kansas; Mexico: Puebla, Morelos, Oaxaca) yielded a total of 49 haplotypes containing 87 polymorphic sites (see Additional file 1). No indels were observed. Of the 12 non-synonymous mutations two (R30K and S291G) are known to be associated with OP resistance [21,22,24] of which the major resistance-associated mutation, S291G, was encountered in all populations examined. A third non-synonymous mutation (Y54H) was present in Europe and in the Colorado population. Five non-synonymous mutations (R30K, L50M, P150S, T151S and A238D) were specific to the Colorado population, one (S55A) to the native Kansas population whereas the remaining four (Q3K, P41S, Y205F and A239T) were specific to the native Mexican populations (see Additional file 2). In general, the number of haplotypes as well as polymorphic sites was lower in Europe than in the US or Mexico (Table 1; see Additional file 1). Notably, the Puebla population yielded most of the polymorphism in Mexico (Table 1) and had many (21 of 37) private substitutions. Although the Mexican Morelos and Oaxaca populations each had only 7 polymorphic sites (Table 1) they possessed 12 private and fixed substitutions. There was only one polymorphic synonymous site present in Europe that was not found in the North American populations. In general, haplotypes were not shared among populations. Only three haplotypes (h1, h4 and h7) were common to both Europe and the US. Three haplotypes (h1, h18, h22) were shared between Colorado and Kansas and five (h42, h43, h45, h46, h49) between Morelos and Oaxaca. The *DP1* and *JHE-b* sequences (n = 91 and 39) yielded 56 haplotypes with 67 polymorphic sites, and 38 haplotypes with 32 polymorphic sites, respectively (see Additional file 1). Similar to *AChE2*, the number of haplotypes and polymorphic sites was, in general, lower in Europe than in North America (Table 1).

**Table 1:** Genetic diversity indices for Colorado potato beetle populations

						**Diversity indices**			
**Population**	**N**	**H**	**S**	**Tr.**	**Pr.**	**k**	**h**	**π**	**θ**_**W**_
	* AChE2* (1037 bp)								
Morelos	20	8	7	0	2	1.979	0.821	0.0019	0.0019
Oaxaca	20	6	7	1	2	2.189	0.800	0.0021	0.0019
Puebla	18	9	37	12	21	10.288	0.843	0.0099	0.0104
Kansas	18	13	30	7	7	10.425	0.948	0.0101	0.0084
Colorado	20	15	38	10	8	8.542	0.963	0.0082	0.0103
Russia	20	5	14	2	0	6.353	0.726	0.0061	0.0038
Finland	20	5	12	2	0	5.795	0.679	0.0056	0.0033
Estonia	20	6	20	4	0	7.021	0.858	0.0067	0.0054
Poland	20	6	20	5	0	7.416	0.790	0.0072	0.0054
Italy	20	4	14	2	0	6.611	0.695	0.0064	0.0038
	* DP1* (931 bp, including a short 58 bp intron site)								
Morelos	20	14	13	3	2	4.684	0.947	0.0050	0.0039
Oaxaca	20	11	14	2	3	4.189	0.916	0.0045	0.0042
Puebla	18	12	22	5	7	6.634	0.915	0.0071	0.0069
Kansas	16	11	25	7	5	8.192	0.942	0.0088	0.0081
Colorado	20	12	34	7	11	6.342	0.905	0.0068	0.0103
Russia	18	4	21	8	0	5.745	0.712	0.0062	0.0066
Finland	20	2	8	2	0	4.211	0.526	0.0045	0.0024
Estonia	10	4	12	5	0	5.311	0.778	0.0057	0.0046
Poland	20	4	12	5	0	5.011	0.753	0.0054	0.0036
Italy	20	4	21	8	0	7.137	0.763	0.0077	0.0064
	* JHE-b* (583 bp)								
Morelos	18	14	12	5	1	4.150	0.974	0.0071	0.0060
Oaxaca	18	11	14	5	3	4.824	0.928	0.0083	0.0070
Kansas	6	2	19	7	5	10.133	0.533	0.0174	0.0143
Russia	14	8	15	6	1	6.044	0.824	0.0104	0.0081
Finland	12	7	7	4	0	3.288	0.864	0.0056	0.0040
Italy	10	4	6	3	0	2.689	0.644	0.0046	0.0036

Abbreviations: N, number of gene copies; H, number of haplotypes; S, number of polymorphic sites; Tr., number of transversions; Pr., number of private polymorphic sites; k, the average number of nucleotide differences; h, haplotype diversity; π, nucleotide diversity; θ_w_, Watterson’s theta estimate.

**Figure 1:**
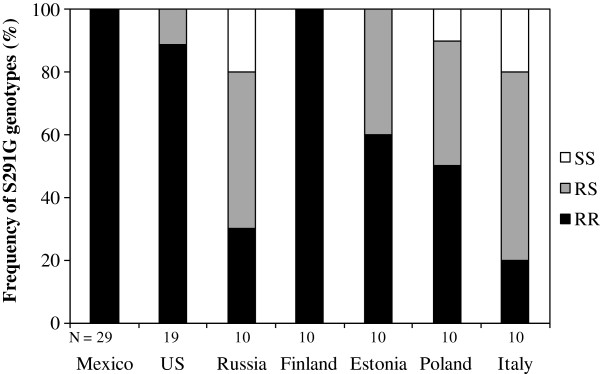
**Frequency (%) of genotypes for the OP resistance-associated mutation S291G in Colorado potato beetle populations.** N; number of individuals, RR; homozygous, RS; heterozygous for the OP resistance-associated mutation S291G, SS: homozygous lacking the S291G mutation.

The frequency of the OP resistance-associated mutation S291G was very high in the US (94.7%) and in Mexican (100%) whereas it was only 71% in the European beetles. At the genotypic level, 100% of genotypes carried the OP resistance-associated mutation S291G in the North America populations and 88.9% (US) and 100% (Mexico) were homozygotes for this mutation (Figure 1). The frequency of genotypes carrying the S291G mutation was 89% in the European populations, and approximately half (52%) of the beetles were homozygous. There were significant differences in the frequencies of genotypes among European populations (*χ*^2^= 16.94, df = 8, P = 0.031). Notably, all individuals in the Finnish population carried S291G whereas the percentage was only 20% in the Italian population (Figure 1). The second resistance associated mutation R30K was found in only two haplotypes (h12, h23) (see Additional file 2) in the Colorado population.

**Figure 2:**
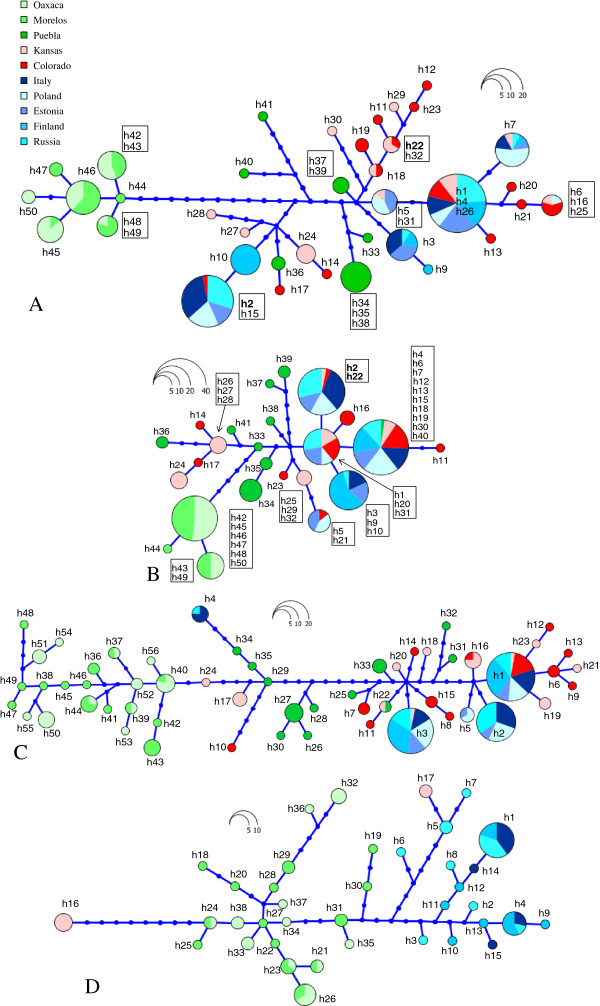
**Haplotype genealogies constructed with HapView.** The circle size is proportional to the haplotype frequency. Each line represents a single mutation; dark blue circles represent extinct haplotypes. **(A)***AChE2* haplotype genealogy based on the portion before the recombination breakpoint recovered by GARD, **(B)***AChE2* haplotype genealogy based on the portion after the recombination breakpoint. Haplotype numbers refer to the *AChE2* haplotypes based on the whole sequences. Haplotypes that lack the S291G mutation are shown in bold. **(C)** Haplotype genealogies for *DP1* and **(D)** for *JHE-b* sequences*.*

Recombination might have played a role in shaping the genetic variation of the *AChE2* locus and may explain the high differentiation among the *AChE2* haplotypes. Indeed, GARD identified a breakpoint (Aic = 5110.7, ΔAic = 237.07) in the alignment between nucleotides 685 and 686 with significant topological incongruence at the p-value = 0.01 while no recombination breakpoints were identified in *DP1* or *JHE-b*. Separate haplotype genealogies for the two portions of the *AChE2* gene were constructed with HapView [41] that uses the topology of most parsimonious trees (DNAPARS, PHYLIP Package) [42] with branch lengths representing haplotype genealogies, i.e. discrete mutational steps. The portion of the gene before the recombination breakpoint contained 37 haplotypes connected by relatively long branches in the haplotype genealogy (Figure 2A). The most common haplotypes in the populations were quite different, separated by 12 mutations. The US and European haplotypes did not form separate clusters but intermingled in the genealogy (Figure 2A). The Mexican Morelos and Oaxaca populations were highly differentiated from other haplotypes, forming a cluster of closely-related haplotypes. The haplotypes that lacked the OP resistance-associated mutation S291G (h22 in US and h2 in Europe) were very different from each other (12 mutational steps, Figure 2A). The portion after the recombination breakpoint showed only 24 haplotypes with the most common haplotypes in the populations being very closely related, differing by only 1 or 2 mutations (Figure 2B).

The LD analysis for *AChE2* indicated that the degree of linkage between sites was considerably lower for the North American populations than in Europe, irrespective of whether sites with minor haplotype frequency (<0.1) were excluded or not (Table 2) . In general, the estimated minimum number of recombination events was higher for the North American populations (Rm = 1–7) than in Europe (Rm = 1–2) with the exceptions of the two Mexican populations, Morelos and Oaxaca, where estimated Rm was 1. The degree of linkage between sites was, in general lower in North America than in Europe for the *DP1* also (Table 2). The estimated minimum number of recombination events was higher for the North American populations (Rm = 1–5) than in Europe (Rm = 0). Finally, the degree of linkage between sites of *JHE-b* was low in both North American and European populations and there were no large differences in Rm either (Table 2).

**Table 2:** Linkage disequilibrium, recombination estimators and neutrality tests for North American and European Colorado potato beetle populations

	**Linkage disequilibrium and recombination**					**Neutrality tests**		
**Population**	**n**	**% sign.**	**% Bonf.**	**R, per gene**	**Rm**	**D**	D*	**ZnS**
	*AChE2* (1037 bp)							
Morelos	21	19.0	0.0	132.0	1	0.010	0.048	0.090
Oaxaca	21	19.0	9.5	18.7	1	0.361	0.674	0.209
Puebla	666	20.6	0.6	4.9	**4**	−0.177	0.057	0.211
Kansas	435	23.4	1.4	16.4	5	0.784	0.042	0.198
Colorado	703	8.3	0.0	7.2	**7**	−0.805	−0.408	0.144
Russia	91	70.3	53.9	1.6	2	**2.232**	0.778	0.575
Finland	66	83.3	37.9	2.5	2	**2.560**	**1.461**	**0.593**
Estonia	190	33.7	9.5	7.9	2	0.933	**1.583**	0.315
Poland	190	27.9	15.3	5.0	1	1.199	1.049	0.350
Italy	91	69.2	25.3	3.7	2	**2.472**	**1.501**	0.524*
	*DP1* (931 bp, including a short 58 bp intron site)							
Morelos	78	12.82	1.28	80.1	5	1.010	0.714	0.140
Oaxaca	91	18.68	3.30	27.6	2	0.226	−0.306	0.135
Puebla	231	14.72	4.33	16.8	2	0.146	−0.523	0.153
Kansas	300	12.67	2.00	10.9	3	0.357	0.206	0.214*
Colorado	561	4.63	1.07	4.8	1	−1.336	−2.144*	0.179
Russia	210	17.14	4.76	0.9	0	−0.231	−1.110	0.412
Finland	28	100.00	100.00	0.001	0	**2.923**	1.342	1.000
Estonia	66	33.33	0.00	7.2	0	1.139	0.856	0.436
Poland	66	34.85	33.33	4.9	0	1.728*	**1.461**	0.438*
Italy	210	55.24	4.76	1.5	0	0.785	**1.592**	0.432
	*JHE-b* (583 bp)							
Morelos	66	12.1	1.5	54.4	4	0.700	**1.460**	0.139
Oaxaca	91	17.6	3.3	18.1	4	0.696	0.803	0.188
Kansas	171	0.0	0.0	0.001	0	1.357	**1.692**	**1.000**
Russia	105	35.2	0.0	6.6	**4**	1.147	0.913	0.317
Finland	21	23.8	4.8	14.1	2	1.637	0.817	0.360
Italy	15	40.0	0.0	0.6	**1**	1.108	1.346	0.668

Abbreviations: n, number of pairwise comparisons;% sign., percentage of significant pairwise comparisons;% Bonf., percentage of significant pairwise comparisons after Bonferroni correction; R, per gene, recombination parameter; Rm, minimum number of recombination events; D, Tajima’s neutrality value; D*, Fu and Li’s neutrality value; ZnS, Kelly’s neutrality value. Significant values in bold. *Significant when recombination was included to the coalescent simulations.

The haplotype genealogy for *DP1* sequences showed quite differentiated haplotypes with long branches connecting them (Figure 2C). The most common haplotypes were separated by 3–8 mutation steps. The US and European haplotypes did not form separate clusters but intermingled in the haplotype genealogy while the Mexican Morelos and Oaxaca haplotypes formed a separate group as in the haplotype genealogy for the portion of the *AChE2* gene before the recombination breakpoint. Although we did not have an extensive sampling for *JHE-b*, the haplotype genealogy was similar in structure to the *DP1* haplotype genealogy (Figure 2D).

### Nucleotide diversity and population genetic structure

There was genetic variation in the *AChE2* gene among the invasive Colorado potato beetle populations (Europe and the Colorado population) (Table 1). Genetic diversity tended to be higher in North America than in Europe for both *AChE2* and *DP1* (Table 1; see Additional file 1 and Additional file 3) but this was statistically significant only for haplotype diversity (Mann–Whitney *U*-test, P = 0.009-0.047). In general, genetic diversity was highest in the US and in the Mexican Puebla populations, and lowest, especially for the *AChE2*, in the other two Mexican populations (Table 1; see Additional file 3). Diversity did not differ among European populations (Kolmogorov-Smirnov test, P > 0.245 for all diversity indices and loci). As for *JHE-b*, there were no significant differences in genetic diversity between North American and European populations (Mann–Whitney *U*-test, P > 0.275 for all diversity indices). There were no significant differences in diversity indices among genes (Kruskal-Wallis test, P > 0.249 for all diversity indices). For Mexican Morelos and Oaxaca populations, diversity tended to be lowest in the *AChE2* gene and highest in the *JHE-b* gene (Table 1; see Additional file 3).

The AMOVA analysis on *AChE2*, *PD1* and *JHE-b* sequences showed significant population genetic structure among regions (Europe, US and Mexico) (P = 0.015-0.024) as well as among populations within regions (P < 0.001-0.045) (Table 3). Among-region variation accounted for most of the genetic variation for *AChE2* (39.86%) and *JHE-b* (56.93%) genes while within-population variation accounted for most of the genetic variation for *DP1* (43.75%). Although, the number of US populations analysed is lower than that of Europe, these differences are large enough that additional sampling would be unlikely to influence our results. No isolation by distance was detected among European populations. Pairwise *Φ*_*ST*_ values for the three loci showed that, in general, the Mexican and the US populations were genetically distinct from European populations, and Mexican populations were genetically distinct from the US populations (Table 4; see Additional file 4). There were few exceptions. There was no significant genetic differentiation between the Colorado and the Estonian populations for *AChE2* (Table 4). And the Kansas and Colorado populations were not genetically different from the Estonian and Italian populations for *DP1* (see Additional file 4). In Mexico, the Puebla population was genetically different from the Morelos and Oaxaca populations while the latter two populations showed no significant genetic differentiation (see Additional file 4). In the US, the Colorado population was genetically different from the native Kansas population (collected from *S. rostratum*) only for *AChE2*. In contrast to North America, the pairwise *Φ*_*ST*_ values showed only a few significant differences among European populations, with the Finnish population being genetically differentiated most often. There was a high correlation between *Φ*_*ST*_statistics among populations with the three genes (r = 0.796-0.987).

**Table 3:** **Analyses of molecular variance (AMOVA) conducted on *AChE2, DP1 *and *JHE-b *sequences of Colorado potato beetle populations grouped together by region (Mexico, US and Europe)**

**Source of variation**	**d.f.**	**Sum of squares**	**Variance components**	**Percentage of variation (%)**	***P***
	*AChE2*				
Among regions	2	484.72	3.385	39.86	0.024
Among populations within regions	7	272.85	1.816	21.38	<0.001
Within populations	186	612.16	3.291	38.76	<0.001
	*DP1*				
Among regions	2	350.13	2.712	41.61	0.023
Among populations within regions	7	140.14	0.954	14.64	<0.001
Within populations	172	490.51	2.852	43.75	<0.001
	*JHE-b*				
Among regions	2	153.17	3.293	56.93	0.015
Among populations within regions	3	11.95	0.115	1.99	0.045
Within populations	72	171.08	2.376	41.08	<0.001

**Table 4:** **Pairwise genetic differentiation (*Φ***_***ST***_**) values for *AChE2 *locus between North American and European Colorado potato beetle populations**

	**Morelos**	**Oaxaca**	**Puebla**	**Kansas**	**Colorado**	**Russia**	**Finland**	**Estonia**	**Poland**
Oaxaca	0.031								
Puebla	**0.754**	**0.752**							
Kansas	**0.742**	**0.736**	**0.239**						
Colorado	**0.788**	**0.782**	**0.322**	**0.064**					
Russia	**0.833**	**0.827**	**0.422**	**0.196**	**0.149**				
Finland	**0.841**	**0.835**	**0.433**	**0.224**	**0.206**	0.086			
Estonia	**0.818**	**0.812**	**0.362**	**0.124**	0.036	0.052	**0.113**		
Poland	**0.808**	**0.802**	**0.366**	**0.122**	**0.086**	0.017	0.122	0.024	
Italy	**0.829**	**0.823**	**0.426**	**0.227**	**0.211**	−0.019	0.062	**0.103**	0.071

Significant *Φ*_*ST*_ values are indicated in bold.

### Neutrality tests

In general, European and the North American populations were in Hardy-Weinberg equilibrium (H-W- test for populations P > 0.05), except for the Mexican Morelos and Oaxaca populations for *AChE2*, Oaxaca and Estonian population for *DP1*, and Oaxaca and Russian populations for *JHE-b* (P < 0.05). In general, the Mexican populations showed no strong deviations from neutrality expectations for all genes (Table 2; see Additional file 3). Tajima’s D and Fu and Li’s D* were mostly close to zero or slightly positive. Neither US population showed strong deviations from neutrality expectations, Colorado had consistently negative and Kansas positive D and D* values. European populations showed some deviation from neutrality expectations, especially for the *AChE2* gene: all neutrality tests were significant for the Finnish and Italian populations while one of the tests was significant for Russia and Estonia (Table 2). All neutrality tests for the *DP1* gene were significant for the Polish population while only one was significant for Finland and Italy. There were no strong deviations from neutrality expectations for the *JHE-b* gene. In contrast to North American populations, most European populations had strong positive neutrality (D and D*) values, indicating an excess of intermediate-frequency variants. An exception was the *DP1* gene for which the Russian population had slightly negative (non-significant) values (Table 2). When European populations were pooled, both Tajima’s D (1.848) and Kelly’s ZnS (0.207) were significant when recombination was included in the coalescence simulations.

## Discussion

Contrary to the expectation that the pattern of genetic variability at the *AChE*2 gene would have been shaped by intense insecticide selection, we found that all genetic variability was associated with the insecticide resistance-associated S291G mutation and that the frequency of this mutation was very high both in the native Mexican and Kansas populations and in the agricultural population in US, though lower in Europe (Figure 1). This lower frequency of S291G among European populations is most likely explained by founder effects associated with the species invasion of Europe (see discussion below) rather than fitness costs of OP resistance in the absence of selection [43]. All three loci showed comparable level of genetic variability with reduced levels of variability in Europe compared to US. There results suggest that pre-invasion history and demographic forces have strongly influenced the evolution of the *AChE2* locus in the invasive Colorado potato beetles.

The high frequency of the insecticide resistance-associated S291G mutation in the native beetle populations together with a high number of substantially differentiated haplotypes (Figure 2A) suggest that the glycine form of AChE2 is the ancestral state. This high frequency of S291G could be related to the native beetles’ adaptation to wild Solanaceous plants which, in general, have higher concentrations of steroidal alkaloids than commercial potato plants [44,45]. Steroidal alkaloids inhibit AChE enzyme and beetle AChE has been shown to be less sensitive to these alkaloids than that of other insects [44]. Thus, most of the observed genetic variation (and the high frequency of S291G) could be related to the beetle’s historical association with alkaloids which caused a pre-adaptation to withstand OP insecticides. Support for this possibility comes from a study on museum samples of blowflies, *Lucilia cuprina,* in which Hartley *et al.*[13] suggested that rapid evolution of OP resistance (malathion) was possible due to a high frequency of resistance alleles before the onset of OP selection.

The unexpected high allelic variation in the *AChE2* gene in addition to the ancestral origin could be due to recombination or to multiple origins of resistance-associated mutations as suggested in the case of *Tribolium castaneum* resistance to cyclodiene insecticide [41]. The recombination analysis indicated several recombination events in both the North American and European samples (Table 2) and GARD indicated the presence of a recombination breakpoint in the middle of the *AChE2* fragment. The portion of the *AChE2* gene after the recombination breakpoint, which is linked to the resistance-associated mutation S291G, showed less allelic variability than the portion before the recombination breakpoint (see Figures 2A and B). This either indicates that selection by OPs could have reduced genetic variability but the variability was subsequentially enhanced by recombination creating the high variability among haplotypes or the portion of the gene linked to the S291G mutation is most constrained. The possibility of multiple origins is difficult to determine since only two susceptible haplotypes have been retained by European and North American populations.

It should be noted that under a scenario in which a beneficial mutation is already relatively common at the onset of positive selection (as it is a likely case in the Colorado potato beetle), the pattern of genetic variation may differ markedly from the classical signature of positive selection [8], not for example, leading to substantial reduction in genetic diversity (e.g. [12]). This may partly explain why no clear signs of insecticide selection at the *AChE2* locus were observed and most allelic variation at the *AChE2* gene in the invasive beetle populations was associated with the resistance-associated mutation. In contrast, for example, in invasive olive fly, *Bactrocera oleae,* the amount of genetic variation of the Mediterranean populations was reduced in areas of high OP usage with two resistant haplotypes reaching proportions above 60% [8].

Although the S291G mutation in the *AChE*2 gene has been associated with OP resistance in the Colorado potato beetle [21,24,45], the intensity of OP selection targeting the S291G mutation may have not been as strong as expected. One reason could be that *AChE2* may not be the major gene conferring OP insecticide resistance. In a recent paper Revuelta *et al.*[46] found that the Colorado potato beetle has a second *AChE* gene that is homologous to the *AChE1* of other insects which in some instance has been found to be the main target of OP selection [47]. Moreover, associations between the resistance S291G mutation and the resistant level of beetle populations have not been very strong [48,49]. Nevertheless, the site-directed mutagenesis has confirmed that the S291G mutation affects the sensitivity of the enzyme to OP insecticides [24] and thus the *AChE*2 gene cannot be totally neutral.

Additionally, negative cross-sensitivity and the fact that the sensitivity of insect AChE to various inhibitors depends on different combinations of several other point mutations at the locus [18] means that selection pressures by OP insecticides and other inhibitors that target AChE may select for different point mutations or combinations of mutations at the *AChE* locus, adding further complexity to the evolutionary history of the locus. For instance, kinetic analyses have indicated that the R30K mutation, in combination with the S291G, enhances AChE2 insensitivity in the beetle [24], whereas a second (I392T) mutation modifies the AChE2 to be susceptible to organophosphates (azinphosmethyl in particular). The exact mechanisms responsible for the mutations’ resistance enhancement/reduction are still unknown. The R30K resistance-associated mutation was very rare in our sample (1%) and found only in Colorado (Table 2) indicating either that the mutation is relatively new or is counter-selected due to the fitness costs [43]. Unfortunately, the *AChE2* sequence we amplified did not include the whole gene, including the I392T mutation, and thus we could not examine the presence of this mutation and other possible mutations elsewhere at the locus. We found another relatively common non-synonymous mutation (Y205F) previously not-described which was fixed in Mexican Morelos and Oaxaca populations. It is unlikely that this mutation affects the function of AChE2 as both tyrosine and phenylalanine have closely similar chemical properties. It is also noted that selection by OPs may have also targeted other resistance mechanisms e.g. enhanced detoxification (reviewed in [29]), which may complicate the situation even further.

The higher frequency of susceptible haplotypes (i.e. serine form of AChE2) among the invasive agricultural populations in Colorado and Europe is intriguing. It could be explained by a relaxation of selection by the original host plant (*S. rostratum*) when the beetle expanded its host range onto cultivated potatoes. In other words, serine form of AChE2 would be the derived form. An interesting scenario, albeit still hypothetical in the light of this data, is that the switch to commercial potato could have counter-selected the glycine form of AChE2, whereas the later usage of OP insecticides could have selected for the S291G mutation restoring the frequency of the ancestral genotype. A historical survey of the frequency of the S291G mutation could reveal whether this hypothesis is true.

The level of genetic variation in the *AChE2* gene (Table 1) was about three times higher than the mtDNA variability in the same populations [34], but similar to that found in the other two nuclear genes, *DP1* and *JHE-b*. There was a high correlation between *Φ*_*ST*_ statistics among populations with the three genes indicating that all three have been affected by similar evolutionary forces. The North American populations were highly genetically differentiated from each other for all three loci examined. The agricultural population in Colorado showed high diversity, similar to the population in Kansas collected on *S. rostratum*. The two populations also shared several haplotypes for all genes analysed suggesting that the switch to potatoes resulted in a large founder population and/or multiple host expansions occurred. The European populations instead showed a strong founder event with a considerable reduction in the genetic variability at all loci (see also [34]). Compared to the North American population, European beetles have lost rare variants and rare mutations (Table 1, Figure 2), which is typical for populations that have experienced a founder event [14]. The bottleneck experienced during the invasion of Europe may also explain the lack of structure among the European populations. In addition to the founder event, the surfing of a few variants on the wave front of the expanding invasive population, reaching relatively high frequencies, could explain the lower genetic variability in Europe [16,50]. These general conclusions are unlikely to be changed by additional sampling. Sampling additional populations in Europe would unlikely lead to an accumulation of additional haplotypes. Although we had only one agricultural population analysed from US, this was the most variable population according to an AFLP and mtDNA survey [34]. Furthermore, our results are also in agreement with those obtained with AFLPs and mtDNA [34] where more populations in US were sampled. Finally, the differences in the distribution of genetic variation among and within regions are large enough that additional sampling would be unlikely to influence our results.

## Conclusions

Examining both native and invasive pest populations can provide important insights on the origins of insecticide resistance mutations and how selection and demographic events modify patterns of genetic variability [10,13]. Here, we have shown that despite the long history of organophosphate (OP) use in the invasive Colorado potato beetle populations, the genetic variability in the *AChE2* gene associated with resistance to OP insecticides has been strongly influenced by the pre-invasion history of the species and demographic events. The high frequency of the S291G mutation in the *AChE2* gene among native populations suggests this mutation is the ancestral state and thus, either a pre-adaptation of the beetle for OP resistance or alternatively *AChE2* is not the main gene conferring OP resistance. The present high genetic variability at the *AChE2* gene could be due to the enduring relationship with host plant alkaloids before the onset of insecticide use and possibly due to recombination events. The genetic diversity in the *AChE2* locus of European beetles strongly reflects founder effects followed by rapid invasion. Our study is a snapshot of the genetic variation as it involves only three genes. It is therefore possible that selection by OP insecticides could show signature elsewhere in the genome. The new omics techniques will allow to test variation as well function of genes and genes network, and thus reveal the effects of insecticide selection at the whole genome level.

## Methods

### Study specimens

Beetles were collected from five regions in Europe: Russia (60°43’N, 33°33’E), Finland (61°41’N, 27°15’E), Estonia (58°23’N, 26°43’E), Poland (53°23’N, 14°32’E) and Italy (45°33’N, 11°33’E) (in all populations n = 10) in 2003. In North America, beetles were collected from two regions in the US: Colorado (40°55’N, 105°07’W) in 2003 and Kansas (38°52’N, 99°19’W) in 2010, and from three regions in Mexico: Puebla (19°01’N, 97°57’W), Morelos (18°47’N, 99°17’W), and Oaxaca (16°43’N, 96°41’W) in 2010 (in all populations n = 10). The Colorado population was chosen for its high levels of genetic variation in AFLP markers [34], and because Colorado presumably was one of the first sites of US invasion [51]. The Kansas and Mexican beetle populations live on the wild relatives of potato, *S. rostratum* (Morelos, Oaxaca and Kansas) and *S. angustifolium* (Puebla). They represent native populations not adapted to use potato as a host [52] and not exposed to insecticide selection. In Mexico, beetles are very host specific [52] and have never been found on potatoes and are not pests of potato [31,53]. Also, the beetle samples collected in Mexico (Morelos, Oaxaca, Puebla) were far from the potato growing regions (Toluca), and thus, unlikely to be exposed to insecticides. Also, the beetles in Kansas were collected from rangelands, where potatoes are not grown.

### DNA analysis

Total genomic DNA was obtained from the legs of beetle individuals. DNA extraction was performed using the DNeasy Tissue Kit (QIAGEN) according to the manufacturer’s protocol. The purification of the lysate was performed with a slight modification from the protocol in which magnetic particles (MagAttract® Suspension G, QIAGEN) were used to shift DNA through purification phases using a Kingfisher (ThermoScientific) automated purification system. Primers used in the amplification of gene sequences were obtained from previously described primers [40,54] as well as designed from the published *AChE2*, *DP1* and *JHE* Colorado potato beetle genes available from Genbank [Genbank: L41180.1, X86074.1]. For primer sequences, PCR mixture and thermal cycle programs, see Additional file 5. ExoI-SAP (Amersham Biosciences) was used to purify the PCR products. Sequencing reactions were performed using the BigDye® Terminator v3.1 Cycle Sequencing Kit (Applied Biosystems) and were run on an ABI 3100 automated sequencer. In general, amplification success was variable. For each of the *AChE2*, *DP1* and *JHE* genes, we were able to obtain sequence data from 98, 91 and 69 individuals of the 100 individuals included in the study, respectively. As for *JHE*, we obtained the *JHE-b* form of the gene [40] for 49 individuals and the *JHE-a* form for 20 individuals. Therefore, only the *JHE-b* form was used in this study.

SeqScape v2.1.1 (Applied Biosystems) was used to assemble and align the sequences. As for *AChE2*, we obtained a 1037 bp long fragment consisting of a short noncoding region [sites 20–109; nucleotide sites were numbered according to the published Colorado potato beetle *AChE* cDNA sequence [55] and a coding region (sites 110–1057) spanning exon 2 to exon 5 of *Drosophila melanogaster AChE*[56]. As for *DP1,* we obtained a 931 bp long fragment consisting of exon 2 [sites 2664–2769; nucleotide sites according to the published Colorado potato beetle *DP1* DNA sequence [38]], a short intron (sites 2770–2827) and part of exon 3 (sites 2828–3594). As for *JHE-b*, we obtained a 1550 bp long fragment (sites 25–1575; nucleotide sites according to [40]. Because the quality of the sequence data for many *JHE-b* samples was poor, we included a 583 bp long fragment (sites 58–641) from 39 individuals (5 populations) in the analyses. No introns were observed in the fragment of the *AChE* and *JHE-b* genes sequenced from the beetle.

Haplotypes were inferred from genotypic data using the program PHASE 2.1.1 [57] which is a Bayesian statistical method for reconstructing haplotypes. Nineteen (*AChE2*) and 17 (*DP1*) individuals, in which the haplotypes could not be inferred reliably by PHASE, were cloned to determine the correct phase of the variable sites. The PCR mix and cycles were as described in Additional file 5 except that the primers used in the amplification of *AChE2* had no M13 tails. The PCR products were purified according to the manufacturer’s instructions using the Qiaquick® PCR purification Kit (QIAGEN) (for *AChE2*) or GeneJET Gel Extraction Kit (Fermentas) (for *DP1*). The ligation and transformation were performed according to the manufacturer’s instructions using Qiagen PCR Cloning Kit (QIAGEN) and Qiagen EZ competent cells (for *AChE2*) or CloneJET PCR Cloning Kit (Fermentas) with NovaBlue Singles cells (Novagen) (for *DP1*). The PCR mixes used to amplify the cloned inserts were as described in Additional file 5 but the primers were M13 for *AChE2* and pJET1.2 for *DP1*. The PCR cycle programs to amplify cloned inserts are described in Additional file 5 and the PCR products were purified and sequenced as described above. Sequences were deposited in GenBank, [GenBank: GU066317 - GU066339, KC342668-KC342788].

### Data analyses

The following diversity indices were calculated using DNASP 4.10.9 [58]: nucleotide diversity (π) [59], allele diversity (h) [59], the average number of nucleotide differences (k) [60] and the Watterson estimator theta (per site) (θ_W_) [61].

To assess whether recombination has had a significant role in shaping genetic variation, we estimated the degree of linkage disequilibrium (LD) by D [62] and its standardized coefficient D’ [63] obtained with the program DNASP. Statistical significance was tested between all pairs of polymorphic sites in each population and for European populations pooled together with the two-tailed Fisher’s exact test [64]. A Bonferroni correction for multiple comparisons was performed [65]. The recombination parameter, R, was estimated by Hudson’s [66] method. The minimum number of recombination events in the history of the sample, Rm, was obtained using the four-gamete test [67]. The confidence limits for Rm were obtained by incorporating recombination in to the simulations implemented in the DNASP (10000 replicates). Finally, we estimated the number and the position of possible breakpoints using the GARD [68] included in the on-line DataMonkey package http://www.datamonkey.org, [69]]. The method screens the multiple-sequence alignments for recombination by searching for evidence of segment-specific phylogenies and assesses goodness-of-fit using an information-based criterion. Departures from Hardy-Weinberg equilibrium were tested in each population with the program ARLEQUIN 3.1 [70]. Haplotype genealogies were constructed with HapView [41]. The parsimonous trees were first constructed with DNAPARS program [PHYLIP package [42]] run from Hapview. Then a consensus tree was constructed with CONSENSE in PHYLIP package. This approach was adopted as phylogenetic reconstruction methods have been shown to perform better than commonly used haplotype network constructing methods for highly genetically divergent haplotype sequence data [41].

We performed a hierarchical analysis of molecular variance (AMOVA) [71] computed with ARLEQUIN to estimate the level of differentiation among populations and between regions. We grouped the populations according to the geographic areas (Mexico, US and Europe), which represent also the different stages of beetle’s invasion. Population pair-wise *Φ*_*ST*_ values for each locus were performed with ARLEQUIN (number of permutations 10000). To test whether genetic distance correlated with geographical distance among European populations, and whether population pair-wise *Φ*_*ST*_ values correlated among populations with genes, a Mantel test was conducted with the program FSTAT 2.9.3 [72] using 10000 randomizations. Geographic distances (kilometers) were entered as log transformed. *Φ*_*ST*_ and Reynold’s distance values for European populations were obtained with ARLEQUIN.

Tests of selective neutrality were analysed with the program DNASP to assess whether the observed frequency distribution of nucleotide polymorphism departs from neutral expectations. Neutrality tests performed included Tajima’s D [73], Fu and Li’s D* [74] and Kelly’s ZnS [75]. The Tajima’s D test compares differences between the number of segregating and the average number of nucleotide differences. It is sensitive to the excess of rare variants (observed after a selective sweep). Fu and Li’s D* test works in a similar manner to the D test where D* is based on the differences between the number of singletons (new mutations) and the total number of mutations, thus, being more sensitive to positive selection than the D test. Kelly’s ZnS statistic tests for the excess of linkage disequilibrium compared with that expected under neutrality. The confidence limits (and the P values) for neutrality test statistics were obtained by coalescent simulations with and without recombination (number of replicates as above).

## Competing interests

The authors declare that they have no competing interests.

## Authors’ contributions

SP, LL, AL, JM and AG designed the study. LL, JM, YHC, VI and AG collected the samples. SP and AG carried out the experimental work, performed the analyses and drafted the manuscript. LL, AL, JM, YHC and VI participated in the draft of the manuscript. All authors contributed and approved to the final version of the manuscript.

## Supplementary Material

Additional file 1 Summary statistics for the Colorado potato beetle populations pooled according to geographical regions. Click here for file

Additional file 2 **Frequencies (%) of observed patterns of non-synonymous mutations of *AChE2 *variants in North American and European Colorado potato beetle populations.** Click here for file

Additional file 3 **Graphical presentation of genetic diversity indices (π, nucleotide diversity; θ**_**W**_, Watterson’s theta estimate) and neutrality tests (Tajima’s D, Fu and Li’s D*) for Mexican, US, and European Colorado potato beetle populations. Click here for file

Additional file 4 **Pairwise genetic differentiation(*Φ***_***ST***_) **for (a) *DP1 *and (b) *JHE-b *loci between European and North American Colorado potato beetle populations.** Click here for file

Additional file 5 **Methodology used in the amplification of *AChE2, DP1 *and *JHE-b *genes.** Click here for file
