# Supplementary material for: Pre-invasion history and demography shape the genetic variation in the insecticide resistance-related acetylcholinesterase 2 gene in the invasive Colorado potato beetle
Source: BMC Evol Biol. 2013 Jan 18;13:13. doi: 10.1186/1471-2148-13-13 (PMC3551707; doi:10.1186/1471-2148-13-13)
Supplement: Additional file 1 — Summary statistics for the Colorado potato beetle populations pooled according to geographical regions. [file 1471-2148-13-13-S1.pdf]

Additional file 1 Summary statistics for the Colorado potato beetle populations pooled according to geographical regions

						Diversity indices						
Region	N	H	S	Tr.	Pr.	k	h	h (SD	π	π (SD)	θ_W	θ_W (SD)
	AChE2 (1037 bp)											
Mexico	58	18	59	17	41	12.540	0.906	0.021	0.0121	0.0013	0.0123	0.0036
US	38	25	45	13	16	9.764	0.967	0.015	0.0094	0.0086	0.0103	0.0033
Europe	100	9	22	5	1	7.009	0.836	0.018	0.0068	0.0023	0.0041	0.0013
Total	196	49	87	25	87	14.048	0.944	0.007	0.0136	0.0005	0.0143	0.0034
	DP1 (931 bp, including a short 58 bp intron site)											
Mexico	58	33	41	10	22	9.154	0.971	0.009	0.0098	0.0007	0.0095	0.0029
US	36	21	41	10	16	7.270	0.946	0.023	0.0078	0.0009	0.0106	0.0035
Europe	88	5	23	10	3	5.576	0.719	0.018	0.0060	0.0005	0.0049	0.0015
Total	182	56	67	18	67	10.837	0.916	0.012	0.0116	0.0004	0.0125	0.0031
	JHE-b (583 bp)											
Mexico*	36	21	15	6	8	4.497	0.957	0.019	0.0077	0.0007	0.0062	0.0024
US (Kansas)	6	2	19	7	5	10.133	0.824	0.098	0.0104	0.0016	0.0081	0.0036
Europe†	36	15	16	7	4	4.514	0.798	0.059	0.0077	0.0010	0.0066	0.0025
Total	78	38	32	13	32	8.732	0.946	0.016	0.0150	0.0002	0.0111	0.0034

Abbreviations: N, number of gene copies; H, number of haplotypes; S, number of polymorphic sites; Tr., number of transversions; Pr., number of private polymorphic sites; k, the average number of nucleotide differences; h, haplotype diversity; π, nucleotide diversity; θ_w, Watterson´s theta estimate. *Morelos and Oaxaca populations, †Russian, Finnish and Italian populations.
