# Supplementary material for: Pre-invasion history and demography shape the genetic variation in the insecticide resistance-related acetylcholinesterase 2 gene in the invasive Colorado potato beetle
Source: BMC Evol Biol. 2013 Jan 18;13:13. doi: 10.1186/1471-2148-13-13 (PMC3551707; doi:10.1186/1471-2148-13-13)
Supplement: Additional file 2 — Frequencies (%) of observed patterns of non-synonymous mutations of AChE2 variants in North American and European Colorado potato beetle populations. [file 1471-2148-13-13-S2.pdf]

Online Supplementary material:

Piiroinen et al. ”*Pre-invasion history and demography shape the genetic variation in the insecticide resistance-related acetylcholinesterase 2 gene in the invasive Colorado potato beetle*”.

Additional file 2 Frequencies (%) of observed patterns of non-synonymous mutations of *AChE2* variants in North American and European Colorado potato beetle populations. S291G and R30K have been associated with organophosphate resistance in the species

			Observed Frequencies (%)									
Pattern	Non-synonymous mutation	Haplotype(s)	Morelos	Oaxaca	Puebla	Kansas	Colorado	Russia	Finland	Estonia	Poland	Italy
a	-	h2,h22	-	-	-	5.6	5	45	-	20	30	50
b	S291G	h1,h4-h7, h10,h13,h15, h16,h18,h20 h21,h24-h29, h31,h32, h33, h36,h37,h39,h41	-	-	38.9	88.9	60	50	85	60	70	30
c	S291G, Y54H,	h3,h9,h17	-	-	-	-	5	5	15	20	-	20
d	S291G, R30K	h12	-	-	-	-	5	-	-	-	-	-
e	S291G, P150S	h11	-	-	-	-	5	-	-	-	-	-
f	S291G, T151S	h14	-	-	-	-	5	-	-	-	-	-
g	S291G, L50M	h19	-	-	-	-	10	-	-	-	-	-
h	S291G, R30K, A238D	h23	-	-	-	-	5	-	-	-	-	-
i	S291G, S55A	h30	-	-	-	5.6	-	-	-	-	-	-
j	S291G, A239T	h34,h35,h38	-	-	55.6	-	-	-	-	-	-	-
k	S291G, Q3K	h40	-	-	5.6	-	-	-	-	-	-	-
l	S291G, Y205F	h42-h47, h50	80	95	-	-	-	-	-	-	-	-
m	S291G, Y205F, P41S	h48,h49	20	5	-	-	-	-	-	-	-	-
