# Supplementary material for: Pre-invasion history and demography shape the genetic variation in the insecticide resistance-related acetylcholinesterase 2 gene in the invasive Colorado potato beetle
Source: BMC Evol Biol. 2013 Jan 18;13:13. doi: 10.1186/1471-2148-13-13 (PMC3551707; doi:10.1186/1471-2148-13-13)
Supplement: Additional file 3 — Graphical presentation of genetic diversity indices (π, nucleotide diversity; θW, Watterson’s theta estimate) and neutrality tests (Tajima’s D, Fu and Li’s D*) for Mexican, US, and European Colorado potato beetle populations. [file 1471-2148-13-13-S3.pdf]

**Online Supplementary material:**
Piiroinen et al. ”*Pre-invasion history and demography shape the genetic variation in the insecticide resistance-related acetylcholinesterase 2 gene in the invasive Colorado potato beetle*”.

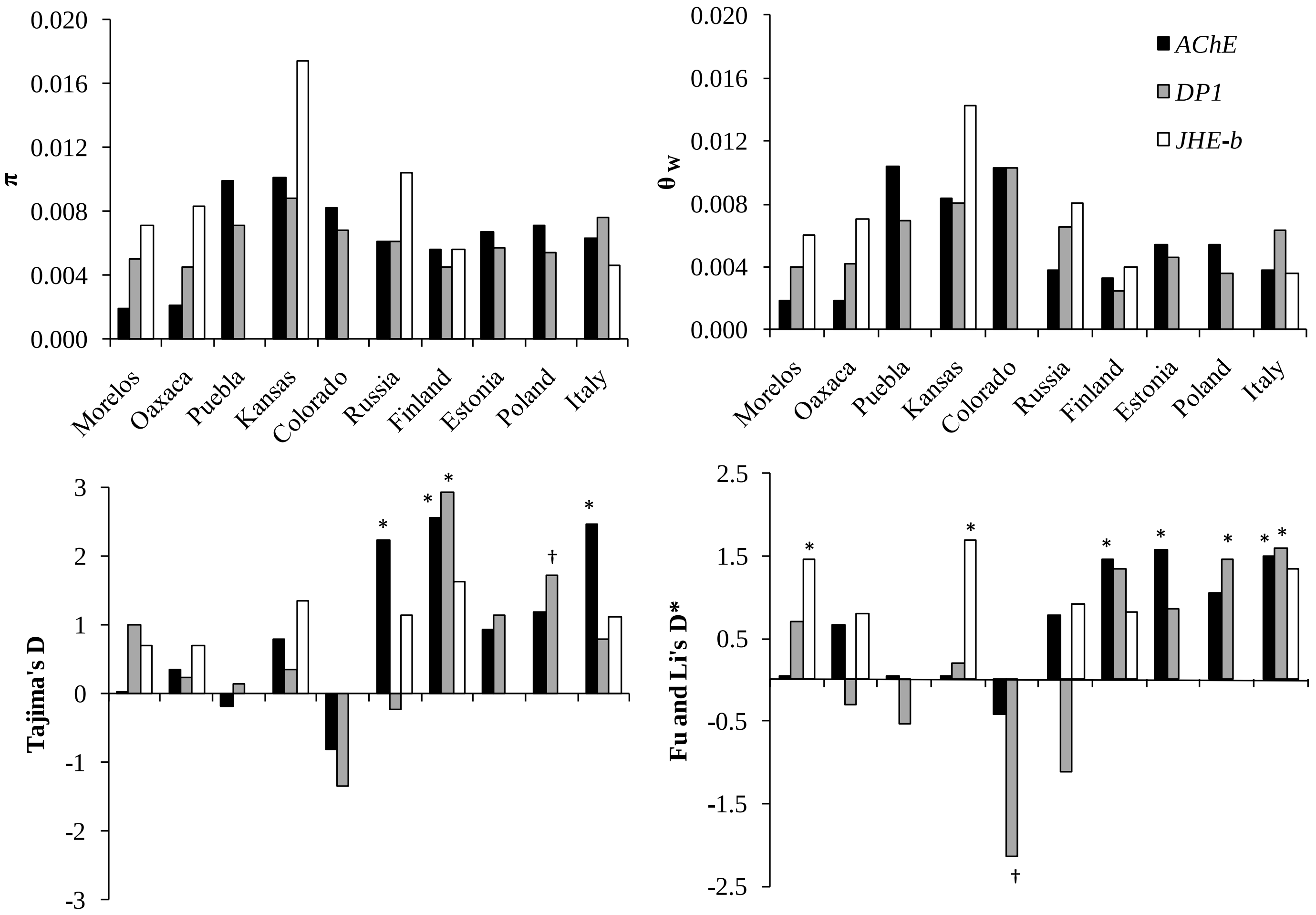


**Additional file 3** Graphical presentation of genetic diversity indices (π, nucleotide diversity, $\theta_W$, Watterson’s theta estimate) and neutrality tests (Tajima’s D, Fu and Li’s D*) for Mexican, US and European Colorado potato beetle populations. * Significant value, †, significant when recombination included to the coalescent simulations
