# Supplementary material for: Pre-invasion history and demography shape the genetic variation in the insecticide resistance-related acetylcholinesterase 2 gene in the invasive Colorado potato beetle
Source: BMC Evol Biol. 2013 Jan 18;13:13. doi: 10.1186/1471-2148-13-13 (PMC3551707; doi:10.1186/1471-2148-13-13)
Supplement: Additional file 4 — Pairwise genetic differentiation(ΦST) for (a) DP1 and (b) JHE-b loci between European and North American Colorado potato beetle populations. [file 1471-2148-13-13-S4.pdf]

Online Supplementary material:

Piiroinen et al. ”*Pre-invasion history and demography shape the genetic variation in the insecticide resistance-related acetylcholinesterase 2 gene in the invasive Colorado potato beetle*”.

Additional file 4 Pairwise genetic differentiation (Φ_{ST}) for (a) *DP1* and (b) *JHE-b* loci between European and North American Colorado potato beetle populations. Significant values are in bold

(a)

	Morelos	Oaxaca	Puebla	Kansas	Colorado	Russia	Finland	Estonia	Poland
Morelos									
Oaxaca	0.029								
Puebla	**0.623**	**0.629**							
Kansas	**0.625**	**0.627**	**0.253**						
Colorado	**0.715**	**0.719**	**0.347**	0.030					
Russia	**0.737**	**0.740**	**0.415**	**0.088**	**0.079**				
Finland	**0.766**	**0.771**	**0.449**	**0.088**	**0.082**	0.096			
Estonia	**0.750**	**0.758**	**0.409**	0.044	0.032	-0.034	-0.030		
Poland	**0.757**	**0.760**	**0.446**	**0.094**	**0.070**	-0.032	0.039	-0.072	
Italy	**0.690**	**0.692**	**0.345**	0.048	0.057	-0.031	**0.107**	-0.002	0.010

(b)

	Morelos	Oaxaca	Kansas	Russia	Finland
Morelos					
Oaxaca	0.004				
Kansas	**0.504**	**0.487**			
Russia	**0.548**	**0.556**	**0.367**		
Finland	**0.663**	**0.660**	**0.477**	**0.108**	
Italy	**0.681**	**0.671**	**0.506**	0.086	0.083
