# Supplementary material for: Pre-invasion history and demography shape the genetic variation in the insecticide resistance-related acetylcholinesterase 2 gene in the invasive Colorado potato beetle
Source: BMC Evol Biol. 2013 Jan 18;13:13. doi: 10.1186/1471-2148-13-13 (PMC3551707; doi:10.1186/1471-2148-13-13)
Supplement: Additional file 5 — Methodology used in the amplification of AChE2, DP1 and JHE-b genes. [file 1471-2148-13-13-S5.pdf]

Additional file 5 Methodology used in the amplification of *AChE2*, *DP1* and putative *JHE* genes. To amplify *AChE2* gene, a forward primer as well as internal primers used in sequencing were designed from the Colorado potato beetle-*AChE2* gene available from Genbank [Genbank: L41180.1] while a previously described primer (Clark *et al.* 2001) was used as the reverse primer. To amplify *PD1* gene, primers were designed from the Colorado potato beetle *DP1* gene available in Genbank [Genbank: X86074.1] while previously described primers (Vermunt et al. 1998) were used to amplify *JHE* gene.

	AChE2	*DP1*	*JHE-b*
Primers			
Forward	5´-*CGACGTTGTAAAACGACGGCCAGT*ACTCAACCCGGTGTTTCA-3´*	5´-CGAATTACCTTAAAAGGGAGCA-3´	5´-ATGGCATCCAATCAAAGATAC-3´
Reverse	5´-*TTTCACACAGGAAACAGCTATGAC*ACTGCTCTCATACAGTCCATCA-3´*	5´-CAAATCTGCTCCAGCTCCAC-3´	5´-GATCATTTTTCAGGTGTCAATTG-3´
Internal forward used in sequencing	5´-AACCTTGGACGTTTACGACG-3	5´-TGAAATCAAGCCCCACTATTT-3´	
Internal reverse used in sequencing	5´-CGTCGTAAACGTCCACCAAGGTT-3	5´-TGGGTGTAGTTTGTCTGAATGG-3´	
PCR mix for amplification (total volume 25µl)	1.5 mM $MgCl_2$, 0.2 mM of each dNTP, 0.2 µM of each primer, 1 x buffer of Taq polymerase (Biotools), 1 unit of Taq DNA polymerase (Biotools) and 20-50 ng of genomic DNA	2 mM MgCl2, 0.2 mM of each dNTP, 0.2 µM of each primer, 1 x buffer of Taq polymerase (Biotools), 1 unit of Taq DNA polymerase (Biotools) and 20-50 ng of genomic DNA	2 mM MgCl2, 0.2 mM of each dNTP, 0.2 µM of each primer, 1 x buffer of GoTaq Flexi polymerase (Promega), 1 unit of GoTaq Flexi DNA polymerase (Promega) and 20-50 ng of genomic DNA
Thermal cycle program	(94°C for 3min) 1 cycle + (94°C for 30s, 60°C for 30s with 1 decrease in temperature per cycle, 72°C for 1min 30s) 10 cycles + (94°C for 30s, 50°C for 30s, 72°C for 1min 30s) 30 cycles + (72°C for 10min) 1 cycle	(94°C for 3min) 1 cycle + (94°C for 1min, 52°C for 1min, 72°C for 1min 30s) 30 cycles + (72°C for 5min) 1 cycle	(94°C for 2 min) 1 cycle + (94°C for 30s, 55°C for 30s, 72°C for 1min 30s) 36 cycles + (72°C for 5min) 1 cycle
Thermal cycle program for amplification of cloned inserts	(94°C for 3min) 1 cycle + (94°C for 30s, 45°C for 30s, 72°C for 1min 30s) 30 cycles + (72°C for 5min) 1 cycle	(95°C for 3min) 1 cycle + (94°C for 30s, 60°C for 30s, 72°C for 1min 30s) 30 cycles + (72°C for 2min) 1 cycle	

*M13-tails in italics
